# The comprehensive interactomes of human adenosine RNA methyltransferases and demethylases reveal distinct functional and regulatory features

**DOI:** 10.1093/nar/gkab900

**Published:** 2021-10-11

**Authors:** Helena Covelo-Molares, Ales Obrdlik, Ivana Poštulková, Michaela Dohnálková, Pavlína Gregorová, Ranjani Ganji, David Potěšil, Lisa Gawriyski, Markku Varjosalo, Štěpánka Vaňáčová

**Affiliations:** Central European Institute of Technology (CEITEC), Masaryk University, Brno 62500, Czech Republic; Central European Institute of Technology (CEITEC), Masaryk University, Brno 62500, Czech Republic; Central European Institute of Technology (CEITEC), Masaryk University, Brno 62500, Czech Republic; Central European Institute of Technology (CEITEC), Masaryk University, Brno 62500, Czech Republic; Central European Institute of Technology (CEITEC), Masaryk University, Brno 62500, Czech Republic; Central European Institute of Technology (CEITEC), Masaryk University, Brno 62500, Czech Republic; Central European Institute of Technology (CEITEC), Masaryk University, Brno 62500, Czech Republic; Institute of Biotechnology & HiLIFE – Helsinki Institute of Life Science, University of Helsinki, Helsinki 00014, Finland; Institute of Biotechnology & HiLIFE – Helsinki Institute of Life Science, University of Helsinki, Helsinki 00014, Finland; Central European Institute of Technology (CEITEC), Masaryk University, Brno 62500, Czech Republic

## Abstract

*N6*-methyladenosine (m^6^A) and *N6*,2′-O-dimethyladenosine (m^6^Am) are two abundant modifications found in mRNAs and ncRNAs that can regulate multiple aspects of RNA biology. They function mainly by regulating interactions with specific RNA-binding proteins. Both modifications are linked to development, disease and stress response. To date, three methyltransferases and two demethylases have been identified that modify adenosines in mammalian mRNAs. Here, we present a comprehensive analysis of the interactomes of these enzymes. PCIF1 protein network comprises mostly factors involved in nascent RNA synthesis by RNA polymerase II, whereas ALKBH5 is closely linked with most aspects of pre-mRNA processing and mRNA export to the cytoplasm. METTL16 resides in subcellular compartments co-inhabited by several other RNA modifiers and processing factors. FTO interactome positions this demethylase at a crossroad between RNA transcription, RNA processing and DNA replication and repair. Altogether, these enzymes share limited spatial interactomes, pointing to specific molecular mechanisms of their regulation.

## INTRODUCTION

Reversible DNA and histone modifications have a well-established role in the regulation of gene expression. RNA modifications, namely *N6*-methyladenosine (m^6^A), were recently recognized as another important layer of regulation ((1,2), reviewed in (3) and (4)). Mammalian cells possess dedicated cellular machinery to write, erase and read m^6^A and m^6^Am (found adjacent to the 5′ cap) marks. Their substrate specificity, regulation and roles in RNA metabolism, development and disease undergo intensive investigations.

In mammals, METTL3 and METTL16 are two m^6^A methyltransferases (MTs) that can modify mRNAs. Although both catalyse the formation of m^6^A in RNA using *S*-adenosylmethionine (SAM) as a methyl donor, they display several rather distinct features. METTL16 appears to act as a monomer (5–7), whereas METTL3 forms a heterodimeric complex with METTL14 (METTL3/14) (8). The METTL3/14 core further assembles with auxiliary proteins that enhance its methylation activity and specificity *in vivo*; WTAP, HAKAI, VIRMA (also known as KIAA1429), RBM15 and ZC3H13 (8–16). METTL16 preferentially methylates structured RNAs carrying a nonameric consensus sequence (UACAGAGAA, the methylated adenosine is underlined) (5,6), whereas METTL3/14 prefers single-stranded RNAs with a short and degenerated motif (DRACH, D = A/G/U; R = A/G; H = A/C/U) (1,2,17).

In mRNAs, the first transcribed adenosines directly adjacent to the 7-methylguanosine (m^7^G) cap can be dimethylated (m^6^Am). The *N6* position is methylated by the cap-specific *N6*-adenosine RNA MT PCIF1 (also known as CAPAM) (18). PCIF1 *N6*-adenosine methylation depends on prior 2′-*O*-methylation of adenosine, as m^6^A was not detected as the first cap-adjacent adenosine (19,20). PCIF1 is recruited to nascent transcripts via direct interaction with Ser5-phosphorylated (Ser5-P) C-terminal domain (CTD) of RNA polymerase II (RNAPII) (18,21).

The levels of m^6^A and m^6^Am in the cells are fine-tuned by the action of at least two demethylases (DMTs): FTO (22) and ALKBH5 (23). FTO was reported to bind the transcription co-activator TRIP4 *in vitro* (24), the protein kinase CaMKII (25) and the tRNA methyltransferase TRMT10A (26). ALKBH5 interacts with two members of the DEAD-box (DDX) family of RNA helicases, DDX46 and DDX3 (27,28). However, the role of most of these interactions and the mechanisms of DMTs regulation remain largely unknown.

The activity of MTs and DMTs determines the *N6*-methylation status of mRNAs and non-coding (ncRNAs) in the cell and, in turn, their metabolism and function. Several pieces of evidence support a model of co-transcriptional m^6^A and m^6^Am dynamics (18,29–35). METTL3 and PCIF1 are recruited to active chromatin by RNAPII (18,21,33) and histone H3 trimethylation at lysine 36 guides m^6^A deposition (34). In turn, m^6^A modulates gene expression via the regulation of histone modifications (36). Therefore, a better understanding of the regulation of MTs and DMTs is key to establish their role in cellular metabolism. To date, the possibility of crosstalk between individual MTs and DMTs and their spatial contacts has not been explored.

To address this question, we employed a proteomic approach and mapped the interaction networks of the key enzymes of the m^6^A and m^6^Am pathways METTL3, METTL16, PCIF1, FTO and ALKBH5 in the human cell line HEK293 T-REx Flp-In (293T). We used the proximity-dependent labelling approach BioID coupled to liquid chromatography-tandem mass spectrometry (LC-MS/MS) (37,38). In this method, the bait protein is fused to a promiscuous biotin ligase derived from *Escherichia coli* BirA (R118G, BirA*) to label proteins *in vivo* in an approximate radius of 10 nm (39), detecting stable and transient protein-protein interactions. Among others, this method has been successfully used to identify protein interactors of other RNA modifying proteins (40).

## MATERIALS AND METHODS

### Preparation of vectors for mammalian expression

To prepare the common backbone for inducible expression of the modified version of BirA (R118G, BirA*), the BirA* sequence was amplified from pcDNA3.1 mycBioID vector (Addgene #35700) with primers flanked by NotI and XhoI restriction enzyme sites. The PCR product was digested and ligated into pcDNA5/FRT/TO™ vector (Invitrogen) containing an N-terminal 3xFlag tag. To create the NLS-BirA*vector, the SV40 nuclear localization signal (NLS) (PKKKRKV) was cloned upstream of the N-terminal 3xFlag tag via KpnI restriction enzyme site. The full-length coding sequences (CDS) of the bait genes were amplified with iProof high-fidelity DNA polymerase (Bio-Rad) from cDNA prepared from 293T cells. For cDNA preparation, total RNA was isolated with TriPure reagent (Roche) according to manufacturer's protocol, treated with Turbo DNase (Ambion) and 2 μg of RNA was used for reverse transcription (RT) with oligo dT primers and SuperScript III RT (Invitrogen). The cDNA was then treated with RNase H (Invitrogen) before being used as a template for PCR. METTL3, METTL16, FTO and ALKBH5 were subcloned by restriction endonucleases. The PCR products were digested and ligated into the pcDNA5/FRT/TO™ vector (Invitrogen) containing the 3xFlag tag-BirA* insert. Two N- and C- terminal BirA* fusion versions were prepared for METTL3. FTO and ALKBH5 have the BirA* tag at the N- terminus and METTL16 at the C- terminus. The PCIF1 construct was prepared by Gateway cloning (Invitrogen). MAC-tag (HA-Strep II-BirA*)-N terminal vector, a gift from Markku Varjosalo (Addgene plasmid # 108078; http://n2t.net/addgene:108078) (38) was used as a destination vector for inducible expression of the BirA* tagged fusion protein. The HA-Strep II-eGFP vector was prepared by Gateway cloning (Invitrogen). pTO-HA-StrepII vector (gift from Markku Varjosalo) was used as a destination vector for inducible expression of eGFP fusion protein for control Strep II tag pull-downs. The C-terminal tagged ALKBH5-Strep II-HA vector was prepared by recloning the CDS of ALKBH5 from the pcDNA5/FRT/TO-Flag-ALKBH5- BirA* to the pcDNA5/FRT/TO™-Strep II tag between the HindIII and KpnI restriction sites. All constructs were verified by Sanger sequencing. The sequences of cloning primers are in Supplementary Table S6.

### Cell culture and stable cell lines preparation

Human 293 Flp-In™ T-REx™ (293T) (Invitrogen) cells were cultured in Dulbecco's modified Eagle's medium (DMEM) supplemented with 10% fetal bovine serum (FBS) at 37°C in the presence of 5% CO_2_. To prepare stable cell lines with inducible expression of the fusion proteins, 293T cells were grown to 70% confluency in 6-well plates format and co-transfected with 300 ng of the corresponding expression vector (pcDNA5/FRT/TO™, Invitrogen, MAC-tag-N or HA-Strep II tag) and 2.7 μg of pOG44 vector (Invitrogen) (ratio 1:9) using 5 μl of TurboFect reagent (Invitrogen) following manufacture's protocol. One day after transfection, cells were transferred to a 150 mm dish and selected in 60 μg/ml of hygromycin B until individual clones were formed. Doxycycline (dox)-inducible expression of the tagged proteins were confirmed by western blot with anti-Flag antibodies (Sigma, 1:5000) or anti-Strep tag II (Abcam, 1:2000) and individual clones were selected.

### Immunofluorescence analysis

Cells were grown on polyethyleneimine-coated coverslips. The expression of BirA* tagged proteins was induced by the addition of 200 ng/ml of dox for 24 h at 37°C. All the subsequent steps were performed at room temperature. Cells were fixed in 3.7% paraformaldehyde for 20 min. Fixed cells were washed with PBS, permeabilized by 0.2% Triton X-100 in PBS for 20 min and blocked for 1 h in 5% horse serum in PBS, then incubated with anti-Flag primary antibody (Sigma, 1:500) or anti-HA (Santa Cruz, 1:100) in 3% horse serum for 1 h. After three washes with PBS for 10 min, cells were incubated with a mix of Alexa 594 secondary antibodies (Invitrogen, 1:500) and DAPI (Sigma, 1:500) in PBS for 30 min in the dark and consequently washed with PBST, PBS and finally fixed on slides in mowiol (Mowiol^®^, Sigma) with DABCO. Samples were imaged with upright microscope Zeiss AxioImager.Z2 combined with Hamamatsu ORCA Flash 4.0 camera. Images were processed using the open-source platform Fiji.

### Biotin-mediated proximity labelling and streptavidin affinity purification (BioID)

Cells were checked for mycoplasma contamination by RT-PCR prior affinity purification (AP) by BioID. PCR primers sequences are in the Supplementary Table S6. For each bait, two 100 mm cell culture dishes were grown to 90% confluency. Twenty-four hours prior cell harvesting the protein expression was induced with 200 ng/ml of dox and 50 μM biotin (Sigma) was added to the medium to allow *in vivo* biotinylation. Cells from the two dishes were combined, pelleted as one biological replicate, flash frozen and short-term stored at -80°C until affinity purification was performed. At least three independent biological replicates were prepared per cell line.

AP was performed according to (41) with minor modifications. Cell pellets were thawed on ice, lysed in 1.2 ml of lysis buffer per frozen pellet and homogenized with a 2 ml syringe and a 21 G × 1–1/2’’ needle followed by three cycles of sonication on ice (5s on, 10s off, amplitude 35, microtip). The insoluble part was removed by centrifugation 16 500 g 4°C 10 min. The supernatants (from two 100 mm cell culture dishes) were incubated with 150 μl of magnetic streptavidin beads (Dynabeads™ MyOne™ Streptavidin C1, Invitrogen) O/N at 4°C and washed according (41). After washing with wash 3 buffer, beads were washed thoroughly three times with 750 μl of 50 mM Tris–HCl pH 8 to remove any detergents present in the sample that would interfere with mass spectrometry analysis. 1/20th of the beads resuspended in 50 mM Tris–HCl were saved for western blot analysis with HRP-conjugated streptavidin (Thermo Scientific, 1:10 000) prior to mass spectrometry analysis.

### Strep II-tag pull-downs

293T HA-Strep II-eGFP, MAC-PCIF1 and ALKBH5-Strep II-HA cell lines were grown in 15 cm dishes under standard cell culture conditions. At a cell-confluency of 60%, transgene expression was induced with 200 ng/μl doxycyclin. Cells were harvested 24 h after induction in ice-cold PBS and immediately pelleted by centrifugation for 5 min at 500 g, 4°C. To remove residual DMEM and fetal calf serum, cell pellets were washed one additional round with ice cold PBS. For total cell lysates preparation, cell pellets from two 15 cm dishes were resuspended in 1.5 ml ice cold lysis buffer [20 mM HEPES–KOH pH 8; 150 mM KCl, 1 mM EDTA, 0.2% IGEPAL, 1 mM DTT, 1× complete Mini (Roche), 15% glycerol], briefly vortexed and placed on ice for 15 min. Cell homogenates were then sonicated on ice using QSonica sonicator Model Q700 [Sonication setting: microtip probe; amplitude: 35%; 10× (1 s on; 9 s off)]. To deplete cell debris and intact cells, crude cell lysates were centrifuged for 20 min at 16 000 g, 4°C. Clarified total lysates were transferred into new clean tubes and placed on ice (20 μl of each lysate were stored at –20°C for western blot analysis, input). For Strep II-tag pull-down, 50 μl StrepTactinXT bead slurry (iba) were pelleted on a magnetic stand. Beads were washed and equilibrated for 40 min at 4°C in lysis buffer. Equilibrated beads were resuspended in 1 ml total lysate (protein concentration ∼2 mg/ml). For pull-downs of ‘intact RNA’ condition (–RNase A), 40 units of placental RNase Inhibitor complex (Biotechrabbit) were added to the lysates and for ‘digested RNA’ condition (+RNase A), lysates were supplemented with 2 μg/ml RNAse A (Thermo). Pull-downs were performed for 4 h at 4°C in rotation. After incubation, the bead-lysate homogenate was placed for 2 min on an ice-chilled magnetic stand and 20 μl of supernatant were stored at −20°C for western blot analysis (FT, unbound fraction). Beads were washed three times in 1.5 ml of ice chilled high-salt wash buffer [20 mM HEPES–KOH pH 8; 350 mM KCl, 1 mM EDTA, 0.2% IGEPAL, 0.1% Na-deoxycholate, 0.05 mg/ml heparin, 1 mM DTT, 1× complete Mini (Roche), 15% glycerol] for 15 min at 4°C on rotation. After the last wash, beads were resuspended in lysis buffer and transferred to a new tube. Beads were washed three times with lysis buffer 10 min at 4°C on rotation. To reduce the content of detergent prior LC–MS/MS analysis, beads were washed four times in 1.5 ml ice cold PBS 5 min at 4°C on rotation. After the last wash, beads were resuspended in 20 μl of PBS. Two μl were kept in −20°C for western blot analysis with Anti-Strep II tag (1:2000, Abcam) and the rest of the beads were frozen in liquid nitrogen and stored at −80°C until LC–MS/MS analysis.

### Protein mass spectrometry sample preparation and data acquisition

Proteins retrieved by streptavidin AP were digested on beads with trypsin and the resulting peptide mixtures were analyzed using RSLCnano system connected to either Orbitrap Fusion Lumos mass spectrometer (Thermo Fisher Scientific) or Orbitrap Elite hybrid spectrometer (Thermo Fisher Scientific). For details, see Supplementary methods.

### Mass spectrometry data analysis

For Limma filtering pipeline, the MS RAW data files were analyzed using the MaxQuant software and further processed using the software container environment (https://github.com/OmicsWorkflows). For SAINT filtering pipeline, the MS RAW data files were searched with Proteome Discoverer 1.4 (Thermo Scientific) and further processed using Significance Analysis of INTeractome (SAINT) - express version 3.6.3 (42). For details, see Supplementary methods.

### DNA damage assays

For siRNA knockdowns, 293T or U2OS cells were seeded in a six-well plate format and transfected with 20 nM FTO siRNAs (ON-TARGET plus FTO SMARTpool, Dharmacon) or non-target control siRNAs (ON-TARGET plus non-targeting pool, Dharmacon) with Lipofectamine RNAiMAX (ThermoFisher) following manufacture's protocol. 48 h after transfection (or 24 h after seeding for WT and FTO KO cells), 2 mM hydroxyurea (HU) or 1 μM camptothecin (CPT) were added to the cell culture medium and cells were collected after the indicated hours. Western blotting was performed with the following antibodies: Anti-FTO (Abcam, ab126605), Anti-γ-H2AX (Sigma, 05-636-I), Anti-tubulin (Sigma, T6074), Anti-histone 3 (Abcam, ab1791), Anti-pSer317 Chk1 (Merk, DR1025), Anti-pRPA(S4/S8) (Bethyl, A300-245A). Quantification of western blot signals were performed using the software ImageJ.

### GO terms analysis

GO terms enrichment analysis was perform using Panther (http://www.pantherdb.org/) with the following parameters: analysis type PANTHER Overrepresentation Test (GO biological process complete, GO cellular component complete or GO molecular function complete), reference list *Homo sapiens* (all genes in the database), test type Fisher's with FDR corrected for multiple tests. Main figures include manually curated terms due to space constraints. Supplementary Table S5 contains the full list of significant (<0.05 FDR) GO terms. Plots were created using ggplot2 R package.

## RESULTS AND DISCUSSION

### Validation of the BioID approach for m^6^A and m^6^Am modifiers

**Figure 1.**
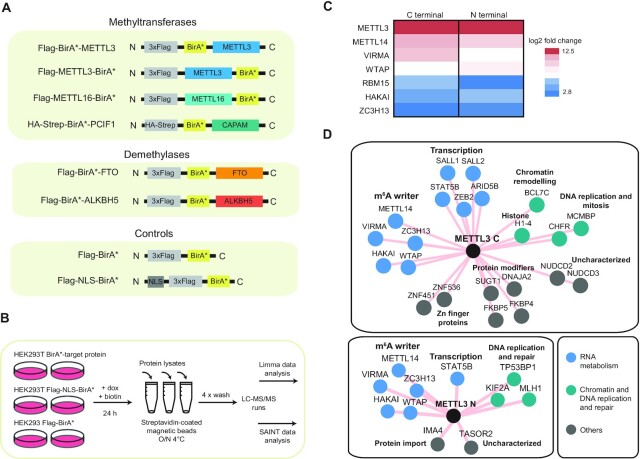
BioID experimental set-up and validation. (**A**) Schematic representation of the BirA*-fusion protein constructs. (**B**) Overview of the BioID experimental design: stable inducible 293T cell lines expressing the BirA*-fusion proteins and the control BirA* and NLS-BirA* were prepared. Fusion protein expression and *in vivo* biotinylation was allowed for 24 h, cell lysates were prepared and biotinylated proteins were isolated by streptavidin-affinity purification. Protein pull-downs were analyzed by LC–MS/MS. Raw MS/MS data were analyzed independently and in parallel by Limma and SAINT analyses. (**C**) Enrichment (log2 fold change of METTL3-BirA* relative to BirA* control) of the known METTL3 complex auxiliary components on METTL3 C- and N- terminal BirA* fusion proteins affinity purifications. All shown protein hits have adjusted *P*-values < 0.01. (**D**) Protein neighbours network of METTL3 identified by BioID. Shared hits from filtered Limma and SAINT data are depicted.

To identify the stable and transient protein interactomes of the key mammalian m^6^A and m^6^Am RNA MTs and DMTs, we performed BioID pull-down assays coupled to LC–MS/MS analysis (37). For each bait, we prepared 293T stable cell lines with inducible expression of BirA* fusion proteins (Figure 1A). All BirA*-fused proteins showed mostly nuclear localization (Supplementary Figure S1A) which is in agreement with previous reports for the endogenous proteins (8,23,30,43–45). As a background control, we used BirA* alone, with mostly cytoplasmic localization, and BirA* fused to a nuclear localisation signal (NLS-BirA*) that targeted BirA* to the nucleoplasm (Figure 1A, Supplementary Figure S1A). Protein lysates of 293T cells expressing the BirA*-fusion proteins were processed in parallel with BirA* and NLS-BirA* controls following the protocol established by Roux el at. (41) with the modifications described in the Methods section (Figure 1B). The biological replicates of each bait displayed good reproducibility and distinct pattern as compared to the other baits (Supplementary Figure S1B). The western blot analysis of proteins precipitated with streptavidin beads indicated efficient enrichment of biotinylated proteins prior to mass spectrometry analysis and already anticipated differences between the individual baits (Supplementary Figure S1C).

### BioID recapitulates the known METTL3 stable interactors and identifies new factors contacting METTL3 *in vivo*

METTL3 is an exception among our bait set, as it has been extensively studied and shown to form a stable complex with other proteins *in vivo*. Therefore, to test whether our BioID approach and analysis of MS/MS can recapitulate known interactors, we first performed LC–MS/MS analysis of the BioID samples from METTL3-BirA* cell lines together with the two background controls, BirA* and NLS-BirA*. To obtain a high-confident list of interacting proteins (hits), the raw MS/MS data were analysed by two independent parallel filtering pipelines—Limma and SAINT (see Materials and Methods and Supplementary Methods). When using the Limma analytical pipeline, only proteins with an enrichment >4-fold and an adjusted *P*-value smaller than 0.01 relative to the control were considered as significant hits. For SAINT we established a SAINT score cut-off of >0.74.

The results from N- and C- terminal BirA* fusions of METTL3 revealed the other essential counterpart METTL14 and all the known auxiliary components of the METTL3/14 complex—WTAP, VIRMA, HAKAI, RBM15 and ZC3H13 - among the top significant Limma hits when BirA* cell line was used as a reference (Figure 1C, Supplementary Figure S2). Importantly, all the known auxiliary components of the METTL3/14 complex except RBM15 were among the shared hits between both filtering pipelines whereas highly probable contaminants were filtered out (Figure 1C, Supplementary Table S1). RBM15 was identified with both N- and C-terminal BirA* fusions of METTL3 by using Limma analysis (Supplementary Table S2). The interaction between RBM15 and METTL3 is mediated by WTAP (9). This likely increases the distance between RBM15 and METTL3 which resulted in less efficient biotinylation of RMB15. Our approach did not detect some of the previously reported METTL3 inetractors, such as SETDB1 and its associated factor TRIM28 previously found in mouse stem cells (46) nor the translation initiation factor eIF3h (47) and TREX mRNA export complex components (48). Nevertheless, we used the combined filtering workflow as it greatly increased confidence while maintaining a high sensitivity of detection. It is also important to point out that due to the nature of the *in vivo* labelling technique used in BioID, we cannot exclude that some of the hits could be biotinylated due to the colocalization and close proximity within the same cellular compartment as the bait proteins, and may not have direct functional consequences.

Apart from the known auxiliary components of the METTL3 complex, our results revealed STAT5B among the top hits in N- and C-terminal METTL3 BioID (Figure 1D, Supplementary Table S1). STAT5B belongs to the STAT (Signal Transducer and Activator of Transcription) family of transcription factors (TFs) which get activated upon binding of cytokines and growth factors to cell surface receptors and then translocate to the nucleus to bind the promoters of their target genes and activate transcription (49). We hypothesize that STAT5B could promote the binding and subsequent methylation of METTL3/14 of certain transcripts, similarly to the reported role of SMAD2/3 in stem cells (50).

### METLL16 BioID detects proteins involved in the biogenesis of RNAs transcribed by the three different RNA polymerases

**Figure 2.**
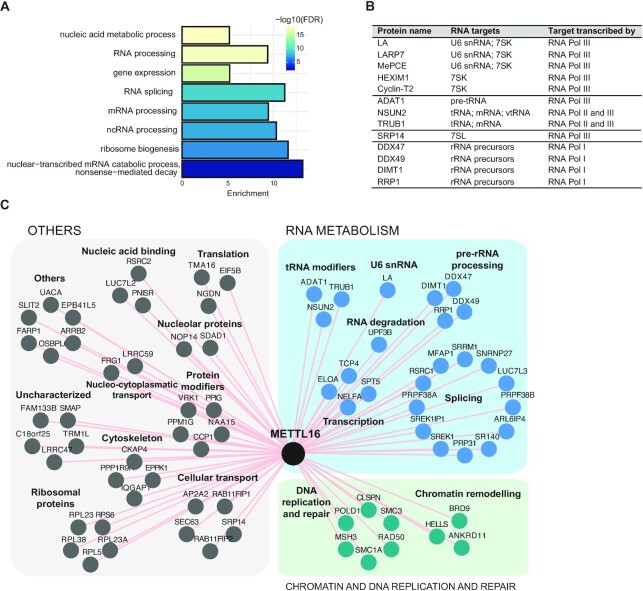
METLL16 BioID reveals proteins involved in the biogenesis of RNAs transcribed by the three nuclear RNA polymerases. (**A**) Representative biological processes (BP) gene ontology (GO) terms significantly enriched among the protein hits identified by METTL16 BioID. (**B**) Protein hits components of RNP particles or involved in the processing and/or modification of RNAs transcribed by RNAPI, II and III. (**C**) Protein neighbors network of METTL16 identified by BioID. Shared hits from filtered Limma and SAINT data are depicted.

We confirmed that our approach can recapitulate known interactors of METTL3 and allows us to obtain highly confident datasets. Therefore, we proceeded to perform the analysis of the other baits. BioID-MS/MS of METTL16, the second human m^6^A MT, identified 78 significant protein hits (Supplementary Table S1). The gene ontology (GO) analysis revealed an enrichment in biological processes (BP) GO terms for mRNA and ncRNA processing and ribosome biogenesis (Figure 2A, Supplementary Table S5). METTL16 targets a wide spectrum of RNAs transcribed by all three RNA polymerases (44), which is reflected in the diversity of METTL16 protein network. Specifically, we identified U6 snRNA biogenesis factors, constituents of 7SK and 7SL particles, tRNA and other ncRNAs modifiers and precursor rRNA processing factors (Figure 2B). METTL16 methylates U6 snRNA at position A43 (5,6,51) and interacts with the early U6 biogenesis factors La, LARP7 and MePCE in an RNA-dependent manner (44), all of which were significant interactors in our dataset (Figure 2B, Supplementary Tables S1, S2). However, LARP7 and MePCE are also stable core components of the 7SK small nuclear ribonucleoprotein (snRNP) particle (52–55) and La protein transiently interacts with 7SK RNA during its biogenesis (54). METTL16 targets 7SK RNA *in vivo* (44). Our BioID experiments revealed additional 7SK snRNP components HEXIM1 and Cyclin-T2 as significant protein hits (Supplementary Tables S2 and S4). The 5′-terminal hairpin of 7SK recognized by HEXIM1 contains triple-based interactions (56) and METTL16 has an affinity to triple-stranded RNA structures (57). Altogether, we hypothesize that METTL16 binding to 7SK could regulate 7SK snRNP assembly and function *in vivo*. We also observed interaction with a component of another RNP particle, the signal recognition particle (SRP) SRP14 (Figure 2B). Notably, 7SL RNA, an RNA component of the SRP, was also identified as a METTL16 substrate (44).

In addition to U6 and 7SK snRNPs, we observed other factors linked to RNAPIII transcripts, the RNA modifying enzymes ADAT1, NSUN2 and TRUB1 (Figure 2B, CSupplementary Tables S1, S4). METTL16 and NSUN2 both target similar types of ncRNAs, such as vault RNAs, Y RNAs, U6 snRNA and lncRNAs (44,58,59). HITS-CLIP analysis of TRUB1 revealed binding to several classes of ncRNAs, but the extent of overlap with METTL16 RNA targets remained unknown as the analysis focused mainly on pri-miRNAs and tRNAs (60). Future studies will address whether the potential interactions between these different modifiers detected by BioID result from their activity on the same RNA molecule or whether they localize to specific nuclear foci that are formed to facilitate RNA processing.

In coding transcripts, METTL16 preferentially binds intronic regions (44). However, so far, only splicing of *MAT2A* transcript was experimentally shown to be regulated by METTL16 activity (51). METTL16 BioID revealed several spliceosome components and splicing factors among the enriched hits (Figure 2C). Among those, SNRNP27 and PRP31 are components and assembly factors of U4/U6.U5 tri-snRNP, respectively (Figure 2C, Supplementary Table S1). It is likely that METTL16 is recruited to pre-mRNAs via binding to U6 snRNPs and subsequently tri-snRNP assembly.

The identified interactors linked to RNAPI include many pre-rRNA processing factors (Figure 2B). Contradictory data have been published to date concerning METTL16 binding to rRNAs (44,57,61). METTL5 and ZCCHC4 were recently identified as 18S and 28S rRNA m^6^A MTs, respectively (62,63). We observed METTL16 interactions with the nucleolar pre-rRNA processing factors DIMT1, DDX47, DDX49 and RRP1 (Figure 2B, C). This agrees with METTL16 localization to the nucleolus (57). Follow up studies will address whether the interaction with these proteins is direct, mediated by pre-rRNAs, or arise from the presence of METTL16 in the nucleolus. In summary, our data indicated that METTL16 is spatially connected to several RNA processing and modification pathways. We hypothesize that cells form distinct subcellular processing and modification bodies assembling specific enzymes to facilitate the processing of diverse coding and ncRNAs.

### PCIF1 BioID detects factors involved in RNAPII transcription initiation and co-transcriptional snRNA biogenesis

**Figure 3.**
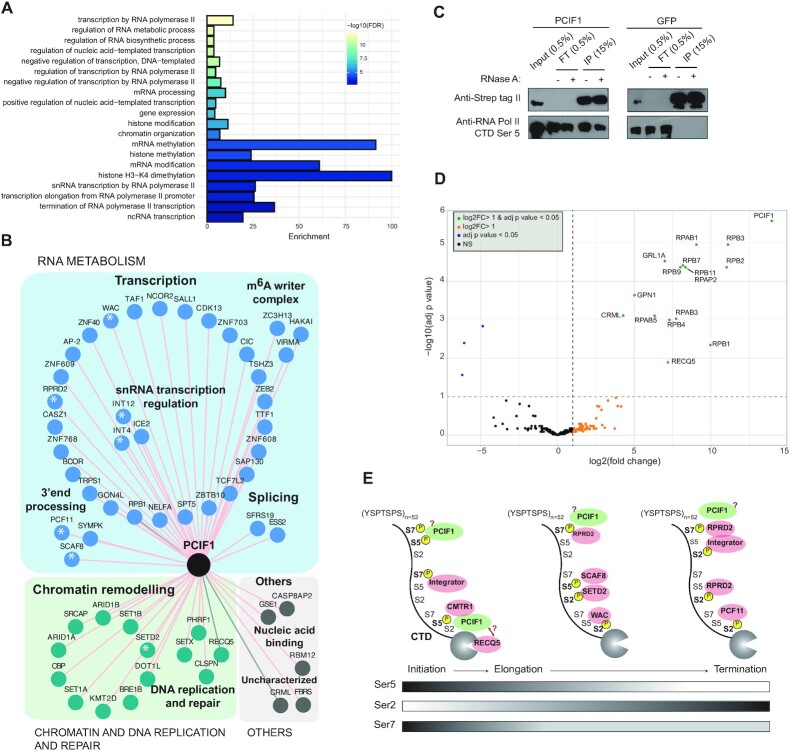
PCIF1 interacts with factors involved in RNAPII transcription initiation and co-transcriptional snRNA biogenesis. (**A**) Representative biological processes (BP) gene ontology (GO) terms significantly enriched among the protein hits identified by PCIF1 BioID. (**B**) Protein neighbours network of PCIF1 identified by BioID. Shared hits from filtered Limma and SAINT data are depicted. Proteins binding RNAPII are marked with an asterisk. Interactions detected also by Strep II-tag pull-downs are connected by grey lines. (**C**) Strep II-tag pull-down of PCIF1 or eGFP fusion proteins from whole-cell extracts from 293T cells. Western blot analysis show efficient pull-down and interaction between PCIF1 and Ser5-phosphorylated RNAPII. FT: unbound fraction. (**D**) Volcano plot representing enrichment (log_2_ fold change) versus significance (–log_10_ adjusted *P*-value) of RNAse A resistant interactions identify in LC-MS/MS analysis of Strep II-tag PCIF1 relative to Strep II-tag eGFP control. Complete list of significant interactions is in Supplementary Table S7. (**E**) RNAPII CTD serine (Ser) phosphorylation pattern during transcription initiation, elongation and termination. Protein hits bound to a specific type of Ser phosphorylation repeat are depicted. The position of RPRD2 on the gene body is only speculative.

PCIF1 is the MT responsible for *N6*-methylation of the 2′-*O*-methylated adenosine that is directly adjacent to the 5′ cap of pre-mRNAs (18,64–66). The GO terms analysis of the 57 hits showed that the most significant BP GO terms were connected with RNAPII transcription (Figure 3A, Supplementary Table S5). Specifically, our BioID results revealed two major links: promoter-proximal pausing and snRNA transcription (Figure 3B). PCIF1 *N6*-adenosine methylation depends on prior ribose 2′-OH methylation by CMTR1 (19,20). We observed an interaction between PCIF1 and CMTR1 indicating that both, the ribose methylation and direct interaction with CMTR1 facilitate PCIF1 activity (Supplementary Table S4). PCIF1, alike the m^7^G mRNA capping enzyme, is recruited to nascent transcripts through specific recognition of Ser5-P CTD of the largest subunit of RNAPII (RPB1) (18,67). We observed RPB1 as the most statistically significant hit in our PCIF1 BioID experiment (Supplementary Table S1).

Except for the largest subunit of RNAPII, other interactors of PCIF1 were not yet tackled. Therefore, in parallel we performed affinity purifications (AP) of Strep II-tagged PCIF1 followed by LC–MS/MS analysis in presence and absence of RNase A (Figure 3C, complete list of significant interactions in Supplementary Table S7). As a background control we used Strep II-tagged eGFP cell line. In agreement with its high affinity to Ser5-CTD of RPB1 (18,21), PCIF1 co-precipitates the whole RNAPII polymerase in an RNA independent manner. Except for RPB6 and 12, we identified the RPB1–12 subunits and GINL1A (Figure 3D and Supplementary Table S7). Other two RNA-independent factors detected by AP and BioID methods were the yet uncharacterized protein CRML and DNA helicase RECQ5 (Figure 3D). In addition, PCIF1 Strep II-AP revealed an RNA independent interaction with two proteins not found in BioID, the Ser2 CTD phosphatase RPAP2 and GTPase GPN1 (Figure 3D and Supplementary Table S7). RECQ5 is a protein with reported roles in transcription and DNA replication and repair (reviewed in (68)). It directly interacts with the cleft of RPB1 of RNAPII and has a negative impact on transcription (69,70).

The BioID PCIF1 interactome revealed more factors involved in transcription regulation. PCIF1 is recruited by RNAPII early during transcription initiation (18), however, RNAPII often pauses early after transcription initiation of protein-coding genes, 20–60 nucleotides downstream of a transcription start site (TSS) (71,72). This promoter-proximal pausing represents an early elongation checkpoint regulated by the negative elongation factor (NELF) and the DRB-sensitivity inducing factor (DSIF) and the Integrator complexes (73). Notably, the PCIF BioID revealed an interaction with SPT5 (of DSIF), NELFA (of NELF) and three components of the integrator complex (INT4, INT6 and INT12) (Figure 3B, Supplementary Tables S1, S3). Importantly, immunofluorescence analysis of PCIF1 revealed that it co-localizes with SPT5 in the nucleoplasm (43). In addition, we observed contacts with several chromatin modifiers responsible for marking transcriptionally active chromatin, such as SETD1A and SETD1B (H3K4me3 MTs) and KMT2D (H3K4me1 MT) (Figure 3B, Supplementary Table S1). It will be interesting to test whether PCIF1 activity plays a role in promoter-proximal stalling of RNAPII and whether the transcripts regulated by this post-initiation mechanism are more frequently carrying m^6^Am at their 5′ termini.

The cap-linked m^6^Am modification was found also in RNAPII transcribed snRNAs and some snoRNAs (74). In this respect, we observed interaction with several factors involved in snRNA synthesis. The BioID results revealed ICE1 and ICE2 subunits of the little elongation complex (LEC), the Integrator and PCIF11 (Figure 3B, Supplementary Tables S1 and S4) and the Strep II AP showed interaction with RPAP2 phosphatase (Figure 3D, Supplementary Table S7). RPAP2 is recruited to snRNA genes via Ser7-CTD of RPB1 and removes the Ser5 CTD marks (75). This may have a positive effect on LEC which regulates snRNA transcription elongation (76). It is possible that crosstalk between PCIF1, RPAP2 and LEC activities facilitates snRNA synthesis. It is also possible that PCIF1 interacts with the NELF complex at the snRNA locus, where it associates with the Integrator and contributes to snRNA transcription elongation and termination efficiency (77). The role of PCIF1 MT activity on snRNAs is yet to be functionally addressed.

The WW domain of PCIF1 shows high homology to the CTD binding WW domain of PIN1 (43), a CTD binding protein that regulates the binding and release of CTD binding factors during RNAPII transcription (78). This could be even uncoupled from its methylation activity as the *Drosophila* PCIF1 homologue is inactive but binds Ser5-P CTD of RNAPII *in vivo* (79). Notably, the list of PCIF1 interactors includes several CTD binding proteins that recognize differently phosphorylated RNAPII CTD, such as the Integrator complex components INT4, INT6 and INT12; CMTR1; SETD2; WAC; SCAF8; PCF11 and RPRD2 (Figure 3B, proteins marked with asterisks; Figure 3E). SETD2 trimethylates H3K36 in the gene body of actively transcribed genes and WAC targets the RNF20/40 ubiquitin-protein ligase complex to active transcription sites to mediate histone ubiquitination (80). Three of these proteins contain CTD-interaction domains (CID): SCAF8, PCF11 and RPRD2. SCAF8, together with SCAF4, suppress the use of early alternative polyadenylation sites on mRNAs (81) and PCF11 is an mRNA and snRNA 3’ end processing factor (82). RPRD2 is known to interact with Ser2-P, Ser7-P and Ser2-Ser7 double phosphorylated CTD, but its function remains unknown (83). PCIF1 likely meets these factors when bound to the CTD, however it is surprising that it occurs in the proximity of factors that recognize CTD modifications present during rather diverse stages of transcription. In summary, our results provided a strong evidence of the role of PCIF1 in the synthesis of snRNAs and suggest a potential role in RNAPII stalling (Figure 3E). It remains an open question whether PCIF1 could also bind Ser7-P or some other forms of modified CTD and whether its recruitment to RNAPII CTD affects the binding of some other CTD interactors.

### FTO on the crossroad between RNAPII transcription, pre-mRNA processing and DNA repair pathways

**Figure 4.**
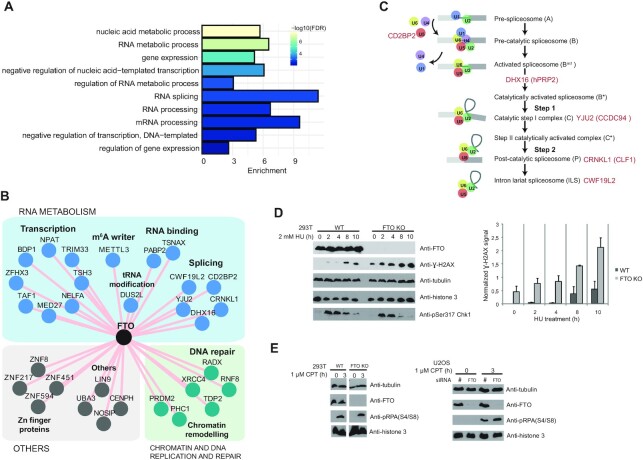
FTO on the crossroad between RNAPII transcription, pre-mRNA processing and DNA repair pathways. (A) Representative biological processes (BP) gene ontology (GO) terms significantly enriched among the protein hits identified by FTO BioID. (**B**) Protein neighbours network of FTO identified by BioID. Shared hits from filtered Limma and SAINT data are depicted. (**C**) Functional states of the fully assembled spliceosome and protein hits identified in FTO BioID. (**D**) 293T *wildtyp*e (WT) and FTO KO cells were treated with 2 mM hydroxyurea (HU) for the indicated hours followed by immunoblotting with antibodies against the indicated proteins (left). Quantification of DNA damage marker γ-H2AX signal normalized to tubulin signal from two independent experiments (mean ± S.D.) (right). (**E**) 293T WT and FTO KO cells (left) and U2OS cells transfected with FTO or non-target (#) siRNAs (right) were treated with 1 μM camptothecin (CPT) for the indicated hours followed by immunoblotting with antibodies against the indicated proteins.

FTO presents a unique feature among the list of enzymes studied in this work as it targets more than one chemical modification. It can demethylate m^6^A, m^6^Am and m^1^A in mRNAs and some ncRNAs *in vitro* and *in vivo* (20,45,84). FTO subcellular localization varies in different cell lines, and its localization was proposed to affect its target specificity (45). The majority of the FTO BioID hits are primarily localized to the nucleus (Supplementary Table S5), which corresponds to FTO nuclear localization in HEK293 (45). The BP GO terms analysis revealed a modest enrichment of general terms connected with transcription, splicing and other RNA metabolic processes (Figure 4A and Supplementary Table S5). The m^6^A and m^6^Am deposition occur mostly co-transcriptionally (18,31–33). The FTO BioID revealed several transcription and chromatin factors indicating that FTO is also recruited to active chromatin loci and acts on nascent RNAs (Figure 4B).

FTO regulates alternative splicing (AS) in mouse and human cells (30,85,86), however, the mechanism of FTO in AS remains unclear. The BioID results support a direct involvement of FTO in pre-mRNA splicing because we observed contacts with spliceosome components and assembly and disassembly factors (Figure 4B, C). Among the most significant were the U5 snRNP 52K protein (CD2BP2) (87), two factors important for the first catalytical step of splicing the DHX16 helicase (yeast *Prp2*) and YJU2 (CCDC94) (88), and CRNKL1 (yeast Syf3) and CWF19L2 (a homolog of yeast Cwf19) (Figure 4C). Interestingly, in yeast, Cwf19 and Syf3 are part of the excised lariat intron U2/U5/U6 complex (89). The presence of spliceosome factors on the proximity of FTO is in agreement with FTO preferential binding to introns (30,45) and suggests co-transcriptional FTO recruitment to nascent pre-mRNA. Furthermore, it raises the possibility that demethylation of pre-mRNA intronic sites directly modulates AS *in vivo* (30). FTO could also affect AS via targeting m^6^Am marks at Sm-class snRNA caps (74). In this respect, the BioID results showed FTO interactions with the snRNA transcription factors SNPC4 and ICE1, components of the SNAPc and little elongation complexes, respectively (Supplementary Tables S2 and S3). Although m^6^Am at snRNAs does not alter snRNP assembly (74), FTO activity may affect AS indirectly through regulating snRNA metabolism for example through pre-snRNA export to the cytoplasm (90).

FTO has the potential to target m^1^A sites in tRNAs (45). A recent study reported that the tRNA m^1^G methyltransferase TRMT10A interacts with FTO and can enhance its catalytic activity *in vitro* (24)*. In vivo* it appears to co-regulate some m^6^A sites in mRNAs (26). The possibility of additional factors mediating the interaction between FTO and TRMT10A was not excluded (24). This could explain why TRMT10A was not enriched on the FTO BioID. However, we observed interactions with three other tRNA modifying enzymes; the dihydrouridine tRNA synthase DUS2L, the guanine dimethyltransferase TRM1 and the PUS TRUB1 (Supplementary Tables S1 and S2). Follow up experiments will tackle the question of whether these enzymes could modulate FTO activity similarly to TRMT10A or whether they simultaneously modify tRNAs.

The most striking result was the identification of proteins involved in DNA replication and repair (Figure 4B), present in FTO and other baits. There is growing evidence of a functional link between m^6^A RNA modification and DNA damage (91–93). m^6^A accumulates at DNA damage sites upon ultraviolet irradiation and, importantly, FTO localizes to laser-induced damage sites in U2OS cells (91). Moreover, m^6^A has been recently detected in R loops, a three-stranded nucleic acid structure formed by a RNA:DNA hybrid and the non-template single-stranded DNA which can be a source of genome instability for the cells (92). Notably, the most enriched and significant FTO BioID interactor was RADX (Supplementary Table S1), a single-strand DNA-binding protein that is recruited to sites of replication stress to promote replication fork stability (94,95). Along with RADX, we observed other factors involved in DNA double-strand break repair (DSB), such as XRCC4, the E3 ubiquitin-protein ligase RNF8 or the Tyrosyl DNA phosphodiesterase 2 (TDP2) (Figure 4B, Supplementary Table S1). To study the functional significance of these interactions we evaluated the effect of treating wildtype and FTO-depleted cells with different types of DNA damaging agents (Figure 4D, E). Similar to RADX depletion (94,95), FTO KO cells displayed accumulation of the DNA damage marker phosphorylated histone H2A variant H2AX (γ-H2AX) upon treatment with 2 mM hydroxyurea (HU) (Figure 4D). No difference between WT and FTO KO or KD cells was seen upon camptothecin (CPT) treatment (Figure 4E). HU slows down the initiation of replication and also the progression of replication forks, whereas CPT blocks DNA synthesis and induces DSBs. These results indicated that FTO could be involved in sensing of specific types of DNA replication stress such as collision between transcription and DNA replication. It remains to be addressed whether the catalytic activity of FTO participates in this process. In summary, FTO appears as a multifunctional protein acting at the intersection between RNA transcription, RNA processing and DNA replication and repair.

### ALKBH5 interacts with pre-mRNA processing and mRNA export factors

**Figure 5.**
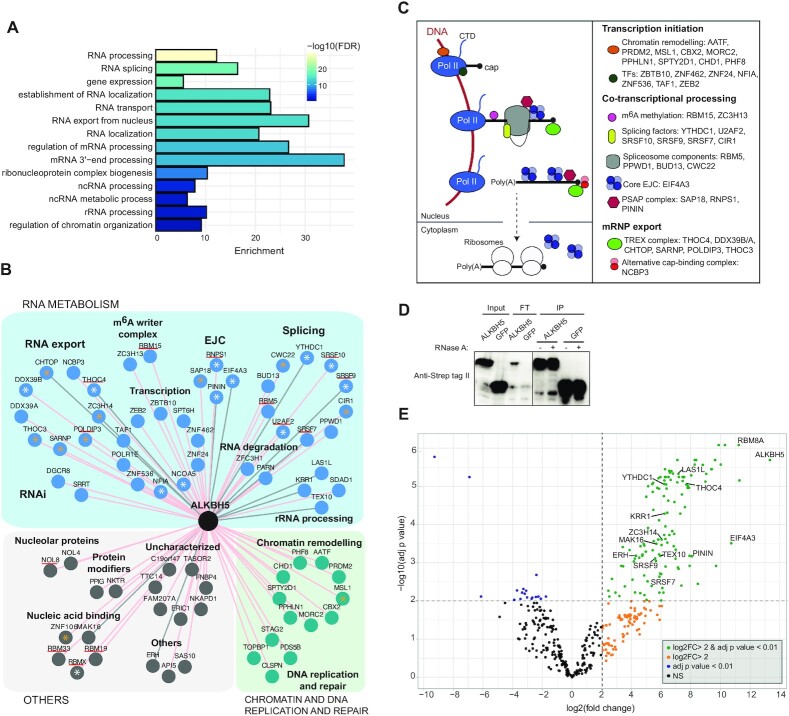
ALKBH5 interacts with mRNA export and pre-mRNA processing factors. (**A**) Representative biological processes (BP) gene ontology (GO) terms significantly enriched among the protein hits identified by ALKBH5 BioID. (**B**) Protein neighbours network of ALKBH5 identified by BioID. Shared hits from filtered Limma and SAINT data are depicted. Proteins reported to localize in the nuclear speckles are marked with an asterisk (white, identified by (112); orange, Uniprot database). Proteins containing an RNA recognition motif domain are underlined in red. Interactions detected also in Strep II-tag pull-downs are connected by grey lines. (**C**) Schematic overview of mRNA co- and post-transcriptional processing (left side). Proteins involved in pre-mRNA processing steps enriched in ALKBH5 BioID are listed (right side). (**D**) Strep II-tag pull-down of ALKBH5 or eGFP fusion proteins from whole-cell extracts from 293T cells. Western blot analysis show efficient pull-down. FT: unbound fraction. (**E**) Volcano plot representing enrichment (log2 fold change) versus significance (–log_10_ adjusted p-value) of RNAse A resistant interactions identify in LC–MS/MS analysis of Strep II-tag ALKBH5 relative to Strep II-tag GFP control. Proteins detected also in BioID analysis are labelled. Complete list of significant interactions in Supplementary Table S7.

ALKBH5 m^6^A DMT was implicated in many parts of mRNA metabolism including splicing regulation, mRNA export and stability of mRNA (23,27,96,97). The BP GO terms enrichment analysis of ALKBH5 *in vivo* interactome reflected connections to nuclear pre-mRNA processing and mRNA export (Figure 5A). In addition, we detected several TFs, as well as proteins involved in chromatin remodeling (Figure 5B) corresponding to the notion that ALKBH5, like PCIF1 and METTL3/14, associates with active chromatin and is co-transcriptionally recruited to the nascent transcripts (35). In contrary to the other baits, ALKBH5 revealed interactions with posttranscriptional mRNA biology (Figure 5B, C). This indicated that ALKBH5 either remains bound to mRNP particles until their export to the cytoplasm or that it is recruited to (pre)mRNA at multiple steps during its biogenesis.

Immunofluorescence analyses showed that ALKBH5 partially co-localizes with nuclear speckle markers in an RNA-dependent manner (23,97). Accordingly, our ALKBH5 BioID data revealed several nuclear speckle proteins (Figure 5B, proteins marked with asterisks). Nuclear speckles are nuclear domains enriched in pre-mRNA splicing factors located in interchromatin regions of the nucleoplasm (98). Among ALKBH5 nuclear speckle interactors, there are several alternative splicing factors and nuclear exon-junction complex (EJC) components (Figure 5B, Supplementary Figure S3A). The assembly of EJC on the mRNA is fully dependent on splicing (99). Specifically, the spliceosomal protein CWC22 interacts with eIF4A3 and serves as a connecting platform between splicing and EJC deposition (Figure 5B,C; Supplementary Figure S3A) (100–102). ALKBH5 also interacted with the nuclear EJC auxiliary complex PSAP (SAP18, RNPS1 and Pinin) (103) (Figure 5B, C; Supplementary Figure S3A).

The top scoring hits in our ALKBH5 BioID were however components of the TRanscription and EXport (TREX) complex. The role of ALKBH5 in mRNA export is supported by an earlier observation that ALKBH5 depletion leads to an accumulation of polyadenylated RNA in the cytoplasm (23). ALYREF (also known as THOC4) showed the highest score followed by the other subunits of TREX complex DDX39B, CHTOP, SARNP, POLDIP3 and the THO subunit THOC3 (Figure 5B, Supplementary Figure S3B, Supplementary Table S1). The TREX complex is recruited to nascent mRNA transcripts during splicing via EJC (104). TREX components, or other export adaptors such as SR proteins, interact with the general mRNA export receptor TAP–p15 complex that transports the mRNP through the nuclear pore. ALYREF and DDX39B accompany the mRNP to the nuclear periphery where they dissociate during translocation through the pore (105). However, the export receptor TAP–p15 complex is not a significant hit in ALKBH5 BioID, indicating that ALKBH5 dissociates from the mRNP at earlier stages of the export process.

Several ALKBH5 BioID interactors possess RNA recognition motifs (Figure 5B, underlined proteins). To further validate the results and assess which of the interactions are RNA independent, we performed Strep II-tag ALKBH5 AP coupled to LC–MS/MS analysis in the presence and absence of RNase A in 293T cells, respectively (Figure 5D). This experiment detected stable, RNA independent interactions with the EJC components eIF4A3, RBM8, MAGOH and Pinin (and Acinus in an RNA-dependent fashion) and the RNA export factors ALYREF and CHTOP (Figure 5E, Supplementary Figure S3, Supplementary Table S7). Other factors found by both analyses were the m^6^A reader and splicing factor YTHDC1 and its interacting partners SRSF9 and SRSF7, the transcription and splicing factor ERH, the ribosome biogenesis factors LAS1L, KRR1, TEX10 and MAK16, and the poly(A) binding and mRNA decay factor ZC3H14 (Supplementary Table S7).

Cooperation between ALKBH5 and TREX components, export adaptors and RNA-binding proteins can modulate mRNA export. This has been shown for two protein hits from ALKBH5 BioID, YTHDC1 and ZC3H14. YTHDC1 is a nuclear m^6^A reader that plays a dual role in the regulation of alternative splicing and mRNA export (106,107). As for ZC3H14, it binds TREX complex components and together ensures the export of a specific subset of mature and properly processed mRNAs in the mouse brain (108). We hypothesize that ALKBH5 could also be an auxiliary factor of the export machinery. For instance, ALKBH5 could remove m^6^A marks to block YTHDC1 mRNA binding. Alternatively, ALKBH5 could play a scaffolding role independent of its demethylase activity. ALYREF was previously identified as a reader of m^5^C mRNA marks deposited by NSUN2 (109). NSUN2 depletion negatively affected ALYREF binding to m^5^C target sites and inhibited the export of NSUN2-modified mRNAs (109). Currently, the mechanism of ALKBH5 and ALYREF interaction in mRNA export is unclear. It is tempting to speculate that ALYREF coordinates the fate of mRNAs depending on its pattern of modifications. In this regard, the first evidence for functional crosstalk between m^5^C and m^6^A was demonstrated in the regulation of p21 expression (110). The 3’ UTR of p21 mRNA bears both m^5^C and m^6^A dependent on NSUN2 and METTL3/14 activities, respectively (110). Silencing of either of these enzymes significantly reduces both modifications concomitantly, and overexpression of NSUN2 and METTL3/14 enhaces p21 protein levels (110). Whether p21 upregulation is due to enhanced mRNA export to the cytoplasm or increased translation was not yet experimentally addressed.

In summary, the interactome analyses revealed that ALKBH5 associates with (pre)mRNAs at multiple levels of their biogenesis (Figure 5C) and strongly indicated the role of ALKBH5 in mRNA export. Furthermore, in the context of other works, it will be exciting to tackle the question of functional crosstalk between different mRNA modifications in gene expression regulation.

### m^6^A and m^6^Am MTs and DMTs have distinctive protein contacts *in vivo*

**Figure 6.**
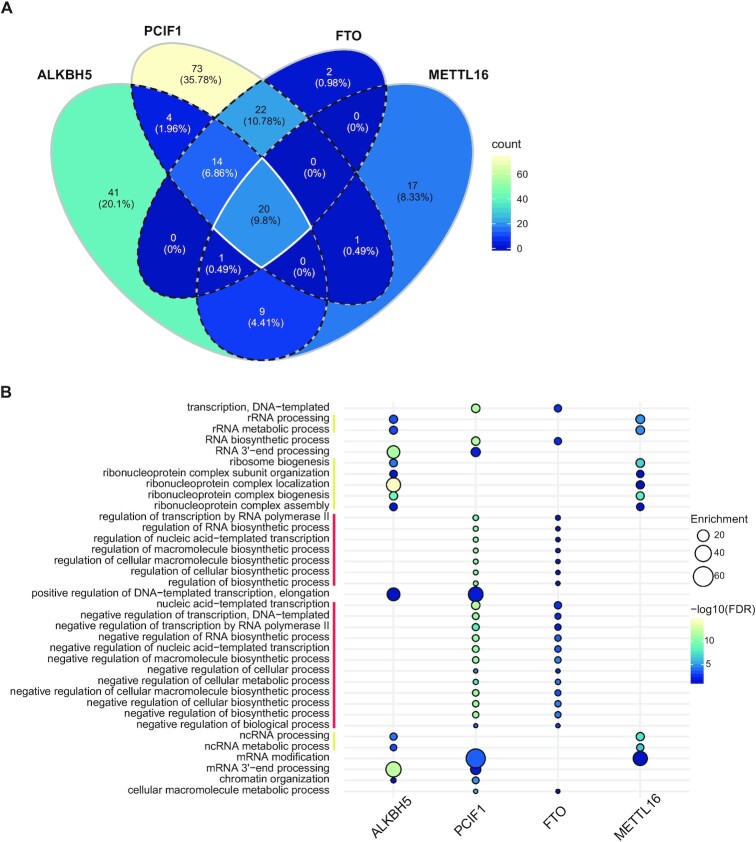
m^6^A and m^6^Am MTs and DMTs have distinctive protein contacts *in vivo*. (**A**) Venn diagram representing the overlap of enriched BP GO terms for the protein hits of the four studied protein baits. The intersection of the four baits is represented with white borders; three baits with white and dashed borders and two baits with all dashed borders. (**B**) Enriched BP GO terms for the protein hits shared by two protein baits. Red, shared by PCIF1 and FTO. Yellow, shared by METTL16 and ALKBH5.

To tackle whether the MTs and DMTs operate or are co-regulated by common factors or pathways, we performed a comparative analysis of the protein networks identified in this study. We first investigated the overlap of enriched BP GO terms between the baits (Figure 6A). The majority of the enriched BP GO terms (65%) were unique for each bait and 9.8% of the GO terms were shared by all four baits (Figure 6A, white borders). The list of 20 shared terms includes different aspects of (m)RNA processing and splicing (Supplementary Figure S4A). ALKBH5 hits showed the highest significance for these terms, reflecting the tighter connection of ALKBH5 with the mRNA processing machinery compared to the other protein baits. In fact, protein domains analysis uniquely identified a significant enrichment of the RNA recognition motif among ALKBH5 protein hits (Figure 5B, underlined proteins). The BP GO terms analysis between pairs of bait proteins (Figure 6A, all dashed borders) revealed the highest overlap between PCIF1 and FTO with 22 shared terms mostly linked to transcription regulation (Figure 6B, terms marked with a red line), which probably corresponds to their shared activity on m^6^Am marks. PCIF1, however, displayed the strongest link with transcription machinery of all the baits tested, revealing a unique enrichment for RNAPII binding, transcription regulation and histone modification (Supplementary Figure S4B, marked with a red line). The second highest overlap between pairs was found between ALKBH5 and METTL16 hits (Figure 6A, all dashed borders) that share nine GO terms, primarily connected with ribonucleoprotein complexes and ncRNA processing (Figure 6B, terms marked with a yellow line). This partial overlap could arise from the nucleolar localization of ALKBH5 and METTL16. Overall, these analyses indicated that mammalian MTs and DMTs possess rather distinctive protein networks, suggesting independent regulation of these enzymes *in vivo*.

Here, we presented a comprehensive analysis of the interactomes of the key RNA adenosine methylases and demethylases. Altogether, our data revealed that these enzymes share a limited number of interactors, pointing to specific molecular mechanisms of their regulation. PCIF1 protein network suggests that it binds nascent RNAs mostly at the transcription loci, whereas ALKBH5 is closely linked to most aspects of pre-mRNA processing and export to the cytoplasm. METTL16 resides in subcellular compartments co-inhabited by several other RNA modifiers and processing factors. FTO interactome points to the role of this DMT at several levels between RNA transcription, RNA processing and DNA replication and repair.

## DATA AVAILABILITY

The mass spectrometry proteomics data have been deposited to the ProteomeXchange Consortium via the PRIDE (111) partner repository with the dataset identifier PXD021566.

## Supplementary Material

gkab900_Supplemental_Files

## Notes

Present address: Helena Covelo-Molares, Center for Research in Molecular Medicine and Chronic Diseases (CiMUS), Universidade de Santiago de Compostela (USC), Santiago de Compostela 15782, Spain.

Present address: Michaela Dohnalkova, Department of Molecular Biology, Science III, University of Geneva, 30 Quai Ernest-Ansermet, 1211 Geneva, Switzerland.

Present address: Pavlina Gregorova, RNAcious Laboratory, Molecular and Integrative Biosciences Research Program, Faculty of Biological and Environmental Sciences, University of Helsinki, Helsinki 00014, Finland.
