# Supplementary material for: The comprehensive interactomes of human adenosine RNA methyltransferases and demethylases reveal distinct functional and regulatory features
Source: Nucleic Acids Res. 2021 Oct 11;49(19):10895–910. doi: 10.1093/nar/gkab900 (PMC8565353; doi:10.1093/nar/gkab900)
Supplement: gkab900_Supplemental_Files [file gkab900_supplemental_files.zip › Supplementary table 6_Revised.docx]

| **Oligo name** | **Sequence (5'-3')** | **Used for** |
| --- | --- | --- |
| BirA_NotI_fwd | ATAGCGGCCGCaaggacaacaccgtgcccctg | Flag-BirA* cloning; FTO -BirA* cloning |
| BirA_XhoI_rev | TATCTCGAGCTActtctctgcgcttctcaggg | Flag-BirA* cloning |
| NLS_KpnI_fwd | CatgCCAAAGAAGAAGCGGAAGGTCggtac | NLS-Flag-BirA* cloning |
| NLS_KpnI_rev | cGACCTTCCGCTTCTTCTTTGGcatGGTAC | NLS-Flag-BirA* cloning |
| METTL3_NotI_fwd | ATAgcggccgcTCGGACACGTGGAGCTCTATC | METTL3-BirA* C and N terminal cloning |
| METTL3_NotI_rev | CCGgcggccgcCTAAATTCTTAGGTTTAGAGAT | METTL3-BirA* C terminal cloning |
| METTL3_XhoI_rev | GCCCTCGAGCTATAAATTCTTAGGTTTAGAG | METTL3-BirA* N terminal cloning |
| FTO_NotI_fwd | GATGCGGCCGCAAGCGCACCCCGACTG | FTO-BirA* cloning |
| FTO_XhoI_rev | CGACTCGAGCTAGGGTTTTGCTTCCAG | FTO-BirA* cloning |
| BirA_NotI_rev | TATGCGGCCGCGcttctctgcgcttctcaggg | FTO-BirA* cloning |
| ALKBH5_KpnI_fwd | ATAGGTACCGCGGCCGCCAGCGGCTACACG | ALKBH5-BirA* cloning |
| ALKBH5_XhoI_rev | TATCTCGAGTCAGTGCCGCCGCATCTTCAC | ALKBH5-BirA* cloning |
| HindIII_ATG_ALKBH5 fwd | AAGAAGCTTATGGCGGCCGCCAGCGGCTACACG | ALKBH5-Strep II tag cloning |
| ALKBH5_KpnI_rev | TATGGTACCTTGTGCCGCCGCATCTTCACC | ALKBH5-Strep II tag cloning |
| METTL16_NotI_fwd | CgcggccgcGCTCTGAGTAAATCaatgcat | METTL16-BirA* cloning |
| METTL16_NotI_rev | AgcggccgcCGTTAACTGCAACAAGCCTGA | METTL16-BirA* cloning |
| PCIF1_NotI_fwd | ATAGCGGCCGCATGGCCAATGAGAATCACG | PCIF1 amplification |
| PCIF1_XhoI_rev | TATctcgagTTAAGTGGGGTGAGGCTCGCGG | PCIF1 amplification |
| PCIF1_attB1_fwd | GGGGACAAGTTTGTACAAAAAAGCAGGCTTCACCATGGCCAATGAGAATCACGGCAG | PCIF1-BirA* cloning |
| PCIF1_attB2_rev | GGGGACCACTTTGTACAAGAAAGCTGGGTTTAAGTGGGGTGAGGCTCGCG | PCIF1-BirA* cloning |
| eGFP_attB1_fwd | GGGGACAAGTTTGTACAAAAAAGCAGGCTTCACCATGGTGAGCAAGGGCGAGGAGCT | eGFP amplification and cloning |
| eGFP_attB2_rev | GGGGACCACTTTGTACAAGAAAGCTGGGTTTACTTGTACAGCTCGTCCAT | eGFP amplification and cloning |
| Myco_fwd | GGCGAATGGGTGAGTAACACG | Mycoplasma contamination testing |
| Myco_rev | CGGATAACGCTTGCGACTATG | Mycoplasma contamination testing |
