# Supplementary material for: The comprehensive interactomes of human adenosine RNA methyltransferases and demethylases reveal distinct functional and regulatory features
Source: Nucleic Acids Res. 2021 Oct 11;49(19):10895–910. doi: 10.1093/nar/gkab900 (PMC8565353; doi:10.1093/nar/gkab900)
Supplement: gkab900_Supplemental_Files [file gkab900_supplemental_files.zip › SupplementaryMaterial_Revised-combined.pdf]

SUPPLEMENTARY FIGURES LEGENDS

Supplementary Figure S1. (A) BirA*-fusion proteins localization by immunofluorescence microscopy. Nucleus stained with DAPI (blue) and fusion proteins with Anti-Flag or Anti-HA (for PCIF1) antibodies (red). Scale bars 24μm. **(B)** Correlation plots of the different biological replicates of BioID experiments for all baits. **(C)** Representative images of streptavidin-affinity purifications. Biotinylated proteins detected with HRP-conjugated streptavidin. FT: unbound fraction.

Supplementary Figure S2. Volcano plots representing enrichment versus significance of METTL3 BioID LC-MS/MS relative to BirA* control. METTL3 C- terminal (left panel) and N-terminal (right panel) BioID protein hits relative to BirA* control. The known auxiliary components of the METTL3/14 complex are indicated. Green dots depict significant protein hits from Limma analysis (full list on Supplementary Table S2).

Supplementary Figure S3. EJC components and export factors interact with ALKBH5 *in vivo*. (A) Core EJC and EJC peripheral proteins. Significant enrichment on BioID and/or Strep II-tag pull-downs is indicated. **(B)** TREX and THO complex components. Significant enrichment on BioID and/or Strep II-tag pull-downs is indicated. **(C)** Strep II-tag pull-down of ALKBH5 or GFP fusion proteins from whole-cell extracts from 293T cells. Western blot analysis show efficient pull-down and RNA-independent interaction between ALKBH5 and ALYREF.

Supplementary Figure S4. Overlap of enriched biological processes (BP) and molecular function (MF) GO terms for the protein hits. (A) Enriched BP GO terms for the protein hits shared by all four protein baits. **(B)** Enriched MF GO terms among the protein hits identified by BioID. Terms unique for each of the protein baits studied are shown.

SUPPLEMENTARY TABLES TITLES

Supplementary Table S1. Proteins identified as significant hits by both Limma (log2 fold change>2 & adjusted p-value<0.01) and SAINT (SAINT score>0.74) analyses.

Supplementary Table S2. Proteins identified as significant hits by Limma analysis (log2 fold change>2 & adjusted p-value<0.01).

Supplementary Table S3. Proteins identified as significant hits by SAINT (SAINT score>0.74) analysis.

Supplementary Table S4. Proteins identified in bait samples and absent from control samples by Limma analysis.

Supplementary Table S5. Full list of significant GO terms.

Supplementary Table S6. PCR primers list.

Supplementary Table S7. Proteins identified in Strep II-tag pull-downs as significant hits by Limma analysis.

SUPPLEMENTARY METHODS

Protein mass spectrometry sample preparation and data acquisition

Following affinity purification washes, bead-bound protein complexes were digested directly on beads by addition of 0.75 µg (1 µg/µl) of trypsin (sequencing grade, Promega) in 50 mM $NaHCO_3$ buffer. Beads were gently tapped to ensure even suspension of trypsin solution and incubated at 37 °C with mild agitation for 2 h. Beads were then vortexed to ensure the release of the partially digested proteins from the beads and the sample was transferred to clean tubes to separate it from the beads and incubated at 37 °C for 16 h without agitation. Resulting peptides were extracted into LC-MS vials by 2.5 % formic acid (FA) in 50 % acetonitrile (ACN) and 100 % ACN with addition of polyethylene glycol (20,000; final concentration 0.001%) (1) and concentrated in a SpeedVac concentrator (Thermo Fisher Scientific).

LC-MS/MS analyses of all peptide mixtures were done using RSLCnano system connected to either Orbitrap Fusion Lumos mass spectrometer (Thermo Fisher Scientific) or Orbitrap Elite hybrid spectrometer (Thermo Fisher Scientific). Prior to LC separation, tryptic digests were online concentrated and desalted using trapping column (100 µm × 30 mm, column compartment temperature of 40 ºC) filled with 3.5-µm X-Bridge BEH 130 C18 sorbent (Waters). After washing of trapping column with 0.1 % FA, the peptides were eluted (flow rate - 300 nl/min) from the trapping column onto an analytical column (Acclaim Pepmap100 C18, 3 µm particles, 75 µm × 500 mm; column compartment temperature of 40 ºC, Thermo Fisher Scientific) by 100 min nonlinear gradient program (1-56% of mobile phase B; mobile phase A: 0.1% FA in water; mobile phase B: 0.1% FA in 80% ACN). Equilibration of the trapping column and the analytical column was done prior to sample injection to sample loop. The analytical column outlet was directly connected to the Digital PicoView 550 (New Objective) ion source with sheath gas option and SilicaTip emitter (New Objective; FS360-20-15-N-20-C12) utilization. ABIRD (Active Background Ion Reduction Device, ESI Source Solutions) was installed.

MS data for ALKBH5, METTL16 and CAPAM baits were acquired on Orbitrap Fusion Lumos mass spectrometer in a data-dependent strategy with cycle time for 3 seconds and with survey scan (350-2000 m/z). The resolution of the survey scan was 60000 (200 m/z) with a target value of 4×10^5 ions and maximum injection time of 50 ms. HCD MS/MS (30% relative fragmentation energy, normal mass range) spectra were acquired with a target value of 5.0×10^4 and resolution of 30 000 (200 m/z). The maximum injection time for MS/MS was 50 ms. Dynamic exclusion was enabled for 60 s after one MS/MS spectra acquisition. The isolation window for MS/MS fragmentation was set to 1.6 m/z.

MS data for FTO and METTL3 baits were acquired on Orbitrap Elite hybrid spectrometer in a data-dependent strategy selecting up to top 10 precursors based on precursor abundance in the survey scan (350-2000 m/z). The resolution of the survey scan was 60 000 (400 m/z) with a target value of 1×10^6 ions, one microscan and maximum injection time of 200 ms. HCD MS/MS spectra were acquired with a target value of 50 000 and resolution of 15 000 (400 m/z). The maximum

injection time for MS/MS was 500 ms. Dynamic exclusion was enabled for 45 s after one MS/MS spectra acquisition and early expiration was disabled. The isolation window for MS/MS fragmentation was set to 2 m/z.

Mass spectrometry data analysis

BioID data analysis

For Limma filtering pipeline, the analysis of the MS RAW data files was carried out using the MaxQuant software (version 1.6.10.43) using default settings unless otherwise noted. MS/MS ion searches were done against modified cRAP database (based on http://www.thegpm.org/crap) containing protein contaminants like keratin, trypsin etc., and UniProtKB protein database for *Homo Sapiens* (ftp://ftp.uniprot.org/pub/databases/uniprot/current_release/knowledgebase/reference_proteomes/Eukaryota/UP000005640_9606.fasta.gz; downloaded 19.09.2018, version 2018/08, number of protein sequences: 21,053). Oxidation of methionine and proline, deamidation (N, Q) and acetylation (protein N-terminus) as optional modification, and trypsin/P enzyme with 2 allowed miss cleavages were set. Only peptides and proteins with FDR threshold <0.01 and proteins having at least one unique or razor peptide were considered. Match between runs was set among all analysed samples. Protein abundance was assessed using protein intensities calculated by MaxQuant.

Protein intensities reported in proteinGroups.txt file (output of MaxQuant) were further processed using the software container environment (https://github.com/OmicsWorkflows), version 3.7.2a. Processing workflow is available upon request. Briefly, it covered: a) removal of decoy hits and contaminant protein groups, b) protein group intensities log2 transformation, c) LoessF normalization, d) differential expression using Limma statistical test. Proteins identified on at least 2 peptides in all biological replicates and passing the threshold of log2 fold change >2 and adjusted p-value < 0.01 were considered as significant hits (Supplementary Table S2). BirA* cell line was used as a background control for the analyses. Proteins identified by at least 2 peptides in all biological replicates in the bait sample and not detected in any of the biological replicates in the matching control sample are listed in Supplementary Table S4.

For SAINT filtering pipeline, the MS RAW data files were searched with Proteome Discoverer 1.4 (Thermo Scientific) using the SEQUEST search engine on the reviewed human proteome in UniProtKB/SwissProt databases (http://www.uniprot.org, downloaded Nov. 2019). Trypsin was selected as the cleavage enzyme and maximum of 2 missed cleavages were permitted, precursor mass tolerance at ±15 ppm and fragment mass tolerance at 0.05 Da. Carbamidomethylation of cysteine was defined as a static modification. Oxidation of methionine and biotinylation of lysine and N-termini were set as variable modifications. All reported data were based on high-confidence peptides assigned in Proteome Discoverer (FDR < 0.01). Then, Significance Analysis of INTeractome (SAINT) -express version 3.6.3 (2) was used to discover statistically significant interactions from the BioID data (cut-off > 0.74) (Supplementary Table S3).

Data filtering

Protein interaction networks were constructed from the shared hits from filtered Limma and SAINT data (Supplementary Table S1) that were imported to Cytoscape 3.7.1. Protein clusters were manually created combining information from several freely accessible databases. The advantage of using the two filtering pipelines is the lower abundance of common contaminants from affinity purification (AP) experiments on the LIMMA+SAINT (double)-filtered set compared to single-filtered sets. To evaluate this, we used the data from multiple negative control AP experiments deposited on the Contaminant Repository for Affinity Purification (the CRAPome) database. We evaluated in how many CRAPome experiments each prey from each of our sets was detected. Occurrence of a given prey on multiple negative control experiments suggested a higher likelihood of being a "false positive". However, the presence of a prey on CRAPome did not exclude it as a "true" interactor. On average, the preys from the double-filtered dataset (Supplementary Table S1) were detected in 7.89% of CRAPome experiments; whereas for LIMMA only (Supplementary Table S2) and SAINT only (Supplementary Table S3) this number increased to 10.90% and 8.53%, respectively. For five out of the six baits studied in this work, the double-filtering pipeline removed more potential contaminants than the single filtering.

Strep II-tag pull-downs analysis

For StrepII-tag pulldowns, Limma filtering pipeline was used with the same parameters used for BioID data. For Strep II-tag ALKBH5 pull-down, proteins passing the threshold of log2 fold change >2 and adjusted p-value < 0.01 were considered as significant hits and for Strep II-tag PCIF1 pull-down log2 fold change >1 and adjusted p-value < 0.05 (Supplementary Table S7). eGFP-Strep II-tag cell line was used as a background control for the analyses.

SUPPLEMENTARY REFERENCES

1. Stejskal, K., Potesil, D. and Zdrahal, Z. (2013) Suppression of peptide sample losses in autosampler vials. *Journal of proteome research*, **12**, 3057-3062.
2. Choi, H., Larsen, B., Lin, Z.Y., Breitkreutz, A., Mellacheruvu, D., Fermin, D., Qin, Z.S., Tyers, M., Gingras, A.C. and Nesvizhskii, A.I. (2011) SAINT: probabilistic scoring of affinity purification-mass spectrometry data. *Nature methods*, **8**, 70-73.

Figure S1

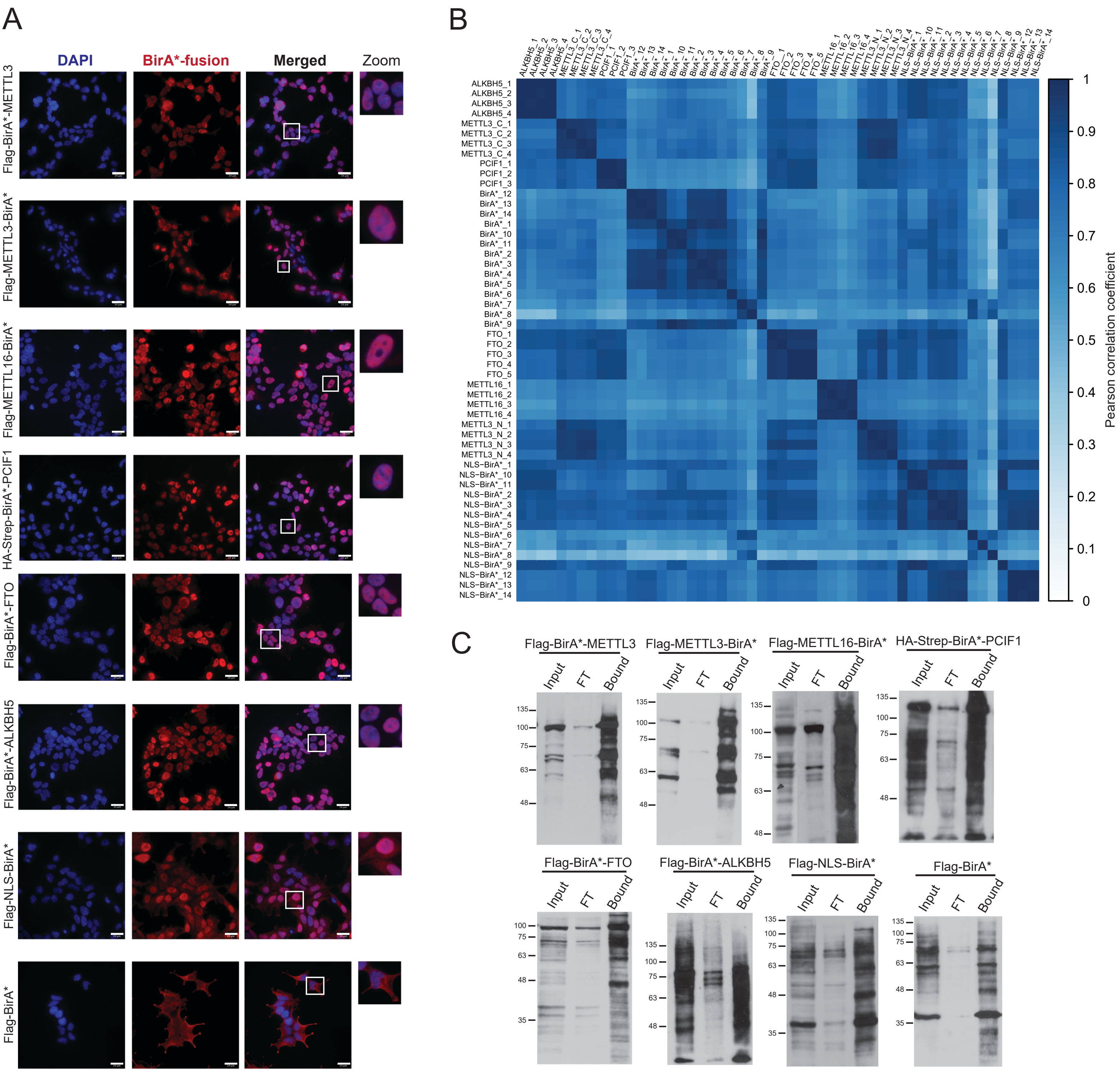
A
DAPI
BirA*-fusion
Merged
Zoom
Flag-BirA*-METTL3
Flag-METTL3-BirA*
Flag-METTL16-BirA*
HA-Strep-BirA*-PCIF1
Flag-BirA*-FTO
Flag-BirA*-ALKBH5
Flag-NLS-BirA*
Flag-BirA*
B
Pearson correlation coefficient
C
Flag-BirA*-METTL3
Flag-METTL3-BirA*
Flag-METTL16-BirA*
HA-Strep-BirA*-PCIF1
Flag-BirA*-FTO
Flag-BirA*-ALKBH5
Flag-NLS-BirA*
Flag-BirA*
Input
FT
Bound

Figure S2

METTL3 C terminal relative to BirA*

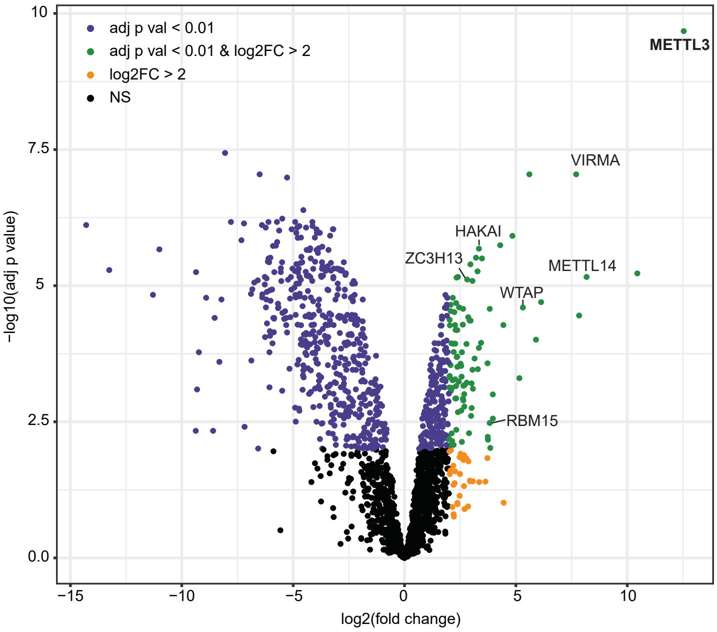

METTL3 N terminal relative to BirA*

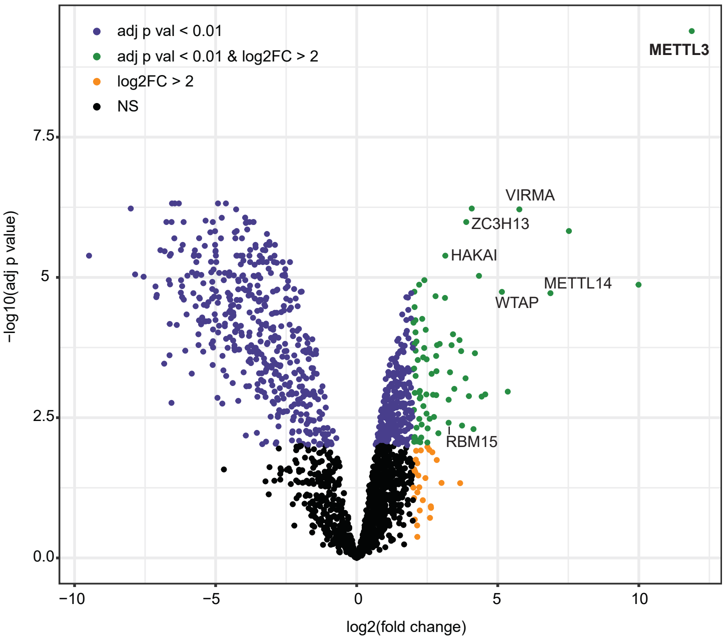

Figure S3

A

Protein name	Cellular localization	EJC-dependent function	Subcomplex	BioID/Strep
**Core EJC**				
eIF4A3	Shuttles; mainly nuclear			Yes/Yes
MAGOH	Shuttles; mainly nuclear			No/Yes
RBM8A/Y14	Shuttles; mainly nuclear			Yes/Yes
BTZ/MLN51	Shuttles; mainly cytoplasmic			No/No
**EJC peripheral proteins**				
RNPS1	Shuttles; mainly nuclear	Splicing; translation; NMD	ASAP; PSAP	Yes/No
SAP18	Shuttles; mainly nuclear	Splicing	ASAP; PSAP	Yes/No
ACINUS	Nuclear	Splicing	ASAP	No/Yes
PININ	Nuclear	Splicing	PSAP	Yes/Yes
ALYREF/THOC4	Shuttles; mainly nuclear	mRNA export	TREX	Yes/Yes
UAP56/DDX39b	Shuttles; mainly nuclear	mRNA export	TREX	Yes/No
URH49/DDX39a	Nuclear	mRNA export	TREX	Yes/No
NXF1	Shuttles; mainly nuclear	mRNA export		No/No
NXT1	Shuttles; mainly nuclear	mRNA export		No/No
UPF3a	Shuttles; mainly nuclear	Translation; NMD		No/No
UPF3b	Nuclear	Translation; NMD		No/No
UPF2	Cytoplasmic	Translation; NMD		No/No
UPF1	Shuttles; mainly cytoplasmic	Translation; NMD		No/No
SMG6	Shuttles; mainly cytoplasmic	NMD		No/No
CWC22	Nuclear	eIF4A3 recruitment to the spliceosome	Spliceosome	Yes/No

B

Protein name	Subcomplex	BioID/Strep
THOC1/hHpr1	THO	No/No
THOC2	THO	No/No
THOC3/hTex1	THO	Yes/No
THOC5/fSAP79	THO	No/No
THOC6/fSAP35	THO	No/No
THOC7/fSAP24	THO	No/No
UAP56/DDX39b	TREX	Yes/No
ALYREF/THOC4	TREX	Yes/Yes
SARNP/CIP29	TREX	Yes/No
CHTOP	TREX	Yes/Yes
POLDIP3	TREX	Yes/No
ZC11A	TREX	No/No

C

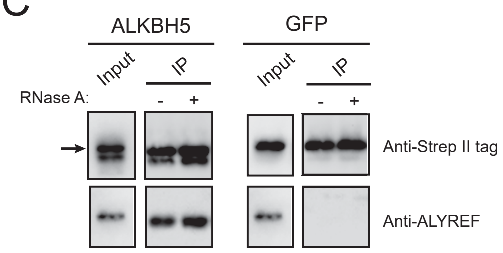

Figure S4

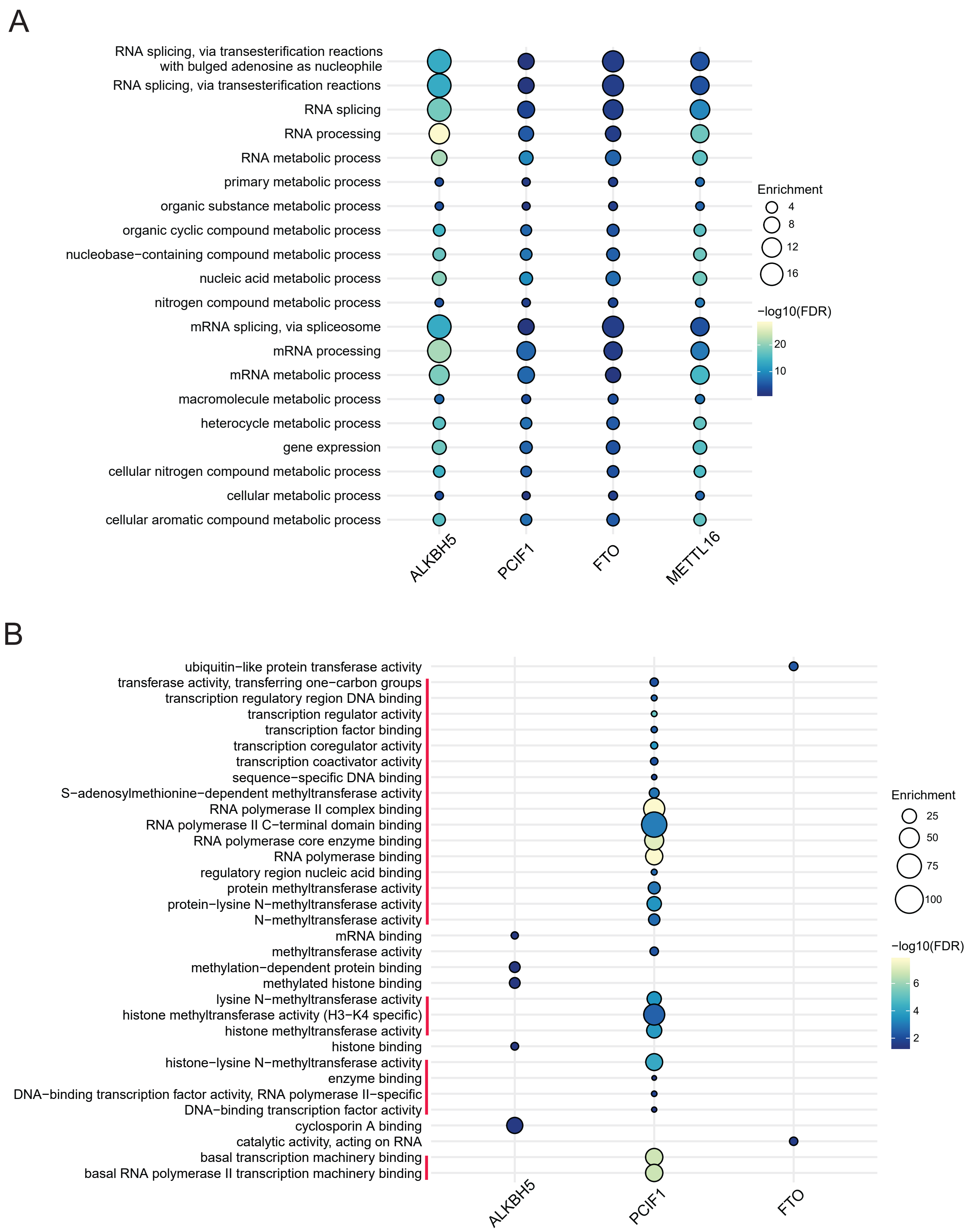
A
RNA splicing, via transesterification reactions with bulged adenosine as nucleophile
RNA splicing, via transesterification reactions
RNA splicing
RNA processing
RNA metabolic process
primary metabolic process
organic substance metabolic process
organic cyclic compound metabolic process
nucleobase−containing compound metabolic process
nucleic acid metabolic process
nitrogen compound metabolic process
mRNA splicing, via spliceosome
mRNA processing
mRNA metabolic process
macromolecule metabolic process
heterocycle metabolic process
gene expression
cellular nitrogen compound metabolic process
cellular metabolic process
cellular aromatic compound metabolic process
ALKBH5
PCIF1
FTO
METTL16
Enrichment
4
8
12
16
−log10(FDR)
20
10
B
ubiquitin−like protein transferase activity
transferase activity, transferring one−carbon groups
transcription regulatory region DNA binding
transcription regulator activity
transcription factor binding
transcription coregulator activity
transcription coactivator activity
sequence−specific DNA binding
S−adenosylmethionine−dependent methyltransferase activity
RNA polymerase II complex binding
RNA polymerase II C−terminal domain binding
RNA polymerase core enzyme binding
RNA polymerase binding
regulatory region nucleic acid binding
protein methyltransferase activity
protein−lysine N−methyltransferase activity
N−methyltransferase activity
mRNA binding
methyltransferase activity
methylation−dependent protein binding
methylated histone binding
lysine N−methyltransferase activity
histone methyltransferase activity (H3−K4 specific)
histone methyltransferase activity
histone binding
histone−lysine N−methyltransferase activity
enzyme binding
DNA−binding transcription factor activity, RNA polymerase II−specific
DNA−binding transcription factor activity
cyclosporin A binding
catalytic activity, acting on RNA
basal transcription machinery binding
basal RNA polymerase II transcription machinery binding
ALKBH5
PCIF1
FTO
Enrichment
25
50
75
100
−log10(FDR)
6
4
2
